# Supplementary material for: PD-L1 on dendritic cells attenuates T cell activation and regulates response to immune checkpoint blockade
Source: Nat Commun. 2020 Sep 24;11:4835. doi: 10.1038/s41467-020-18570-x (PMC7518441; doi:10.1038/s41467-020-18570-x)
Supplement: Supplementary file 1 — Supplementary Information [file 41467_2020_18570_MOESM1_ESM.pdf]

Supplementary Information

PD-L1 on dendritic cells attenuates T cell activation and regulates response to immune checkpoint blockade

Peng et al.

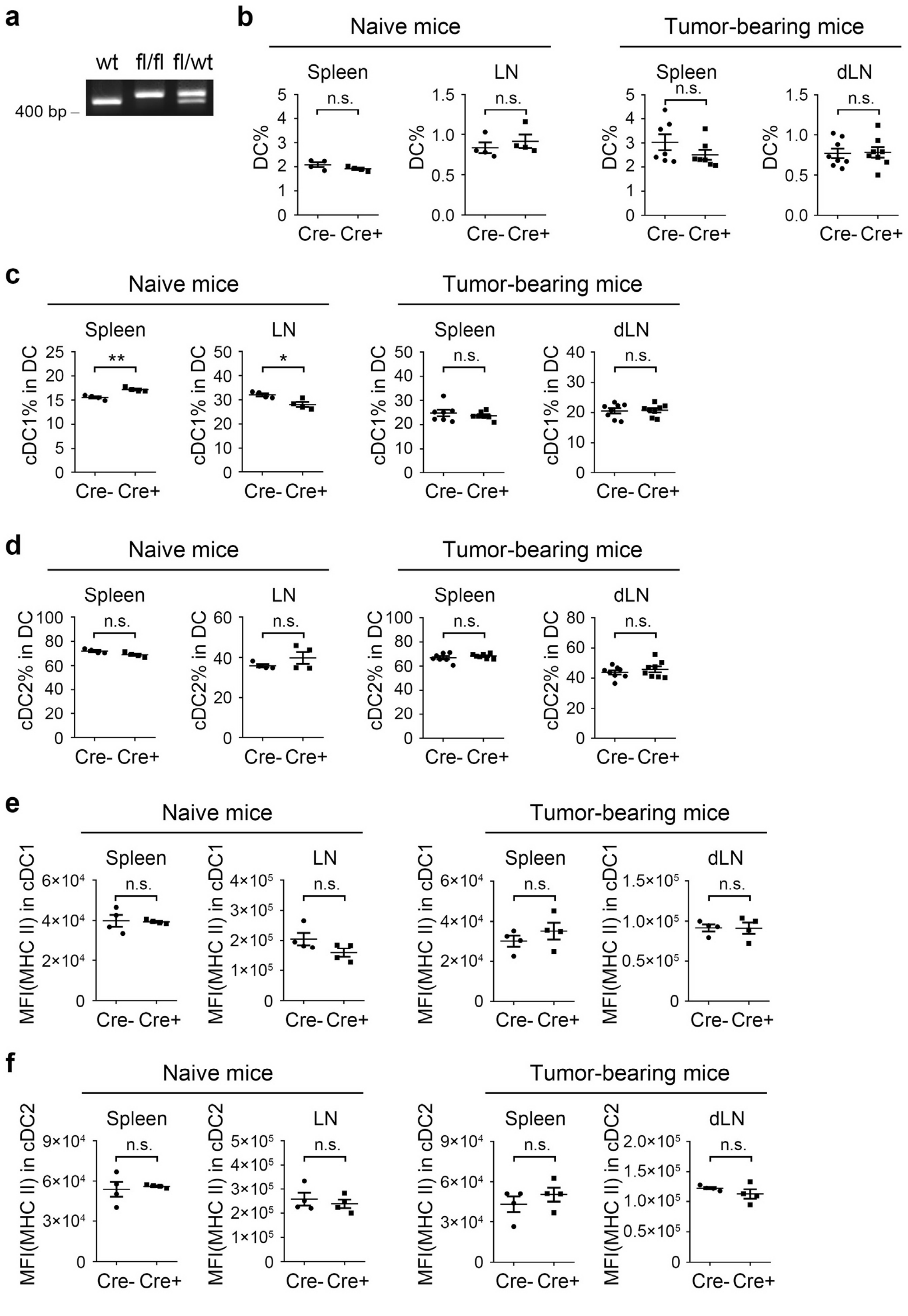

Supplementary Figure 1. Characterization of PD-L1-conditional knockout mice. (**a**) PCR results of genotyping. Data is representative of more than three independent experiments. (**b-f**)

Spleen and LN tissues were collected from naïve or MC38 tumor-bearing conditional knockout mice (for b-d: n = 4 naïve, 7 tumor-bearing spleen, 8 tumor-bearing LN; for e-f: n = 4). Percentages of (**b**) total DCs ($CD11c^{+}MHC\ II^{+}$), (**c**) cDC1 ($CD11b^{-}CD24^{+}$), (**d**) cDC2 ($CD11b^{+}CD24^{-}$), MHC II levels on (**e**) cDC1 and (**f**) cDC2 were measured by flow cytometry. **p=0.0021; *p=0.0158. Data are shown as mean ± SEM and are representative of two independent experiments or pool of two independent experiments. n.s., not significant; *, p<0.05; **, p<0.01 determined by unpaired two-tailed Student's t-test. Source data are provided as a Source Data file.

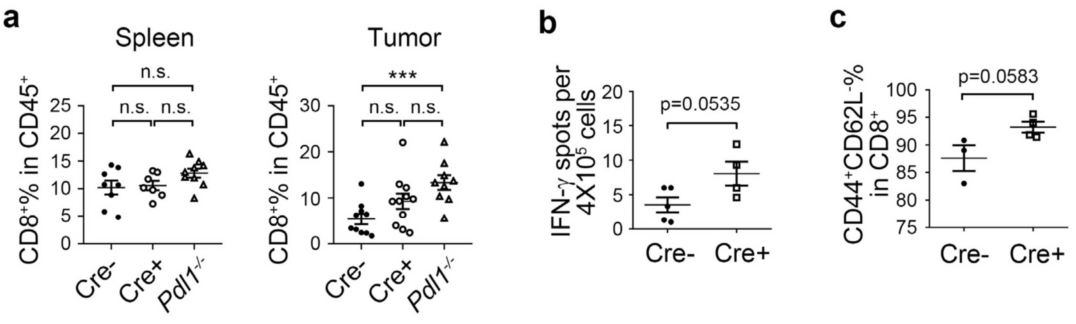

Supplementary Figure 2. PD-L1 on DCs dampens spontaneous antitumor immune responses. CD11c-cre;*Pdl1*$^{fl/fl}$ or control mice were inoculated with MC38 tumor and analyzed on day 14 after inoculation. (**a**) Percentages of CD8^{+} T cells in spleen (n = 8 Cre-, 7 Cre+, 9 *Pdl1*$^{-/-}$) and tumor (n = 10 Cre-, 11 Cre+, 9 *Pdl1*$^{-/-}$) tissues were shown. ***p=0.0007. (**b**) IFN-γ^{+} cells in spleen were measured by ELISPOT (n = 5 Cre-, 4 Cre+). (**c**) CD8^{+} T cell activation in tumor tissues were measured by flow cytometry (n = 3 Cre-, 4 Cre+). Data are shown as mean ± SEM and are representative of two independent experiments (**b** and **c**) or pool of two independent experiments (**a**). n.s., not significant; ***, p<0.001 determined by unpaired two-tailed Student's t-test. Source data are provided as a Source Data file.

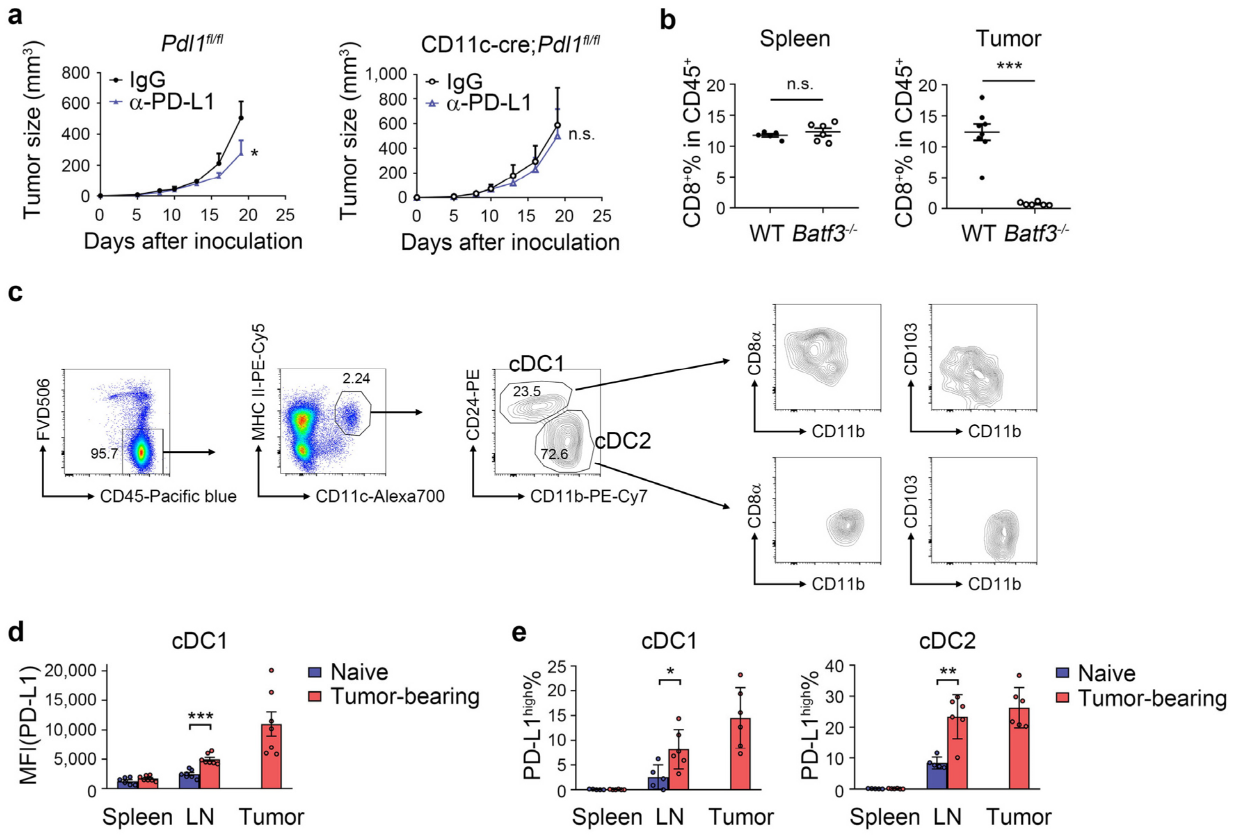

Supplementary Figure 3. PD-L1 on cDC1 plays important roles in antitumor immunity. (**a**) E.G7 tumors established in CD11c-cre;*Pdl1*$^{fl/fl}$ or control mice (n = 4) were treated with IgG or anti-PD-L1 on day 10 and 13. Tumor growth curves were shown. *p=0.0200. (**b**) WT or *Batf3*$^{-/-}$ mice were inoculated with 5×10^5 MC38 cells. Spleen (n = 5 WT, 6 *Batf3*$^{-/-}$) and tumor (n = 8 WT, 6 *Batf3*$^{-/-}$) tissues were collected on day 14 after inoculation and analyzed by flow cytometry. Percentages of $CD8^+$ cells among $CD45^+$ cells were shown. ***p<0.0001. (**c**) Gating strategy for DC subsets. Gated cDC1 and cDC2 were further stained for CD8α and CD103. (**d**) Tissues were collected and analyzed as in **Fig. 3b**. Mean fluorescent intensities (MFIs) of PD-L1 were shown (n = 7 mice, except naïve spleen n = 6). ***p<0.0001. (**e**) In naïve or E.G7 tumor-bearing WT mice (n = 5 naïve, 6 tumor-bearing), PD-L1 levels on cDC1 and cDC2 were measure by flow cytometry. *p=0.0228; **p=0.0013. Data are shown as mean ± SEM or + SEM (**a**) and are

representative of two independent experiments (**a**, **c**, and **e**) or pool of two independent experiments (**b** and **d**). n.s., not significant; *, $p<0.05$; ***, $p<0.001$ determined by two-way ANOVA in (**a**) or by unpaired two-tailed Student's t-test in (**b**, **d**, and **e**). Source data are provided as a Source Data file.

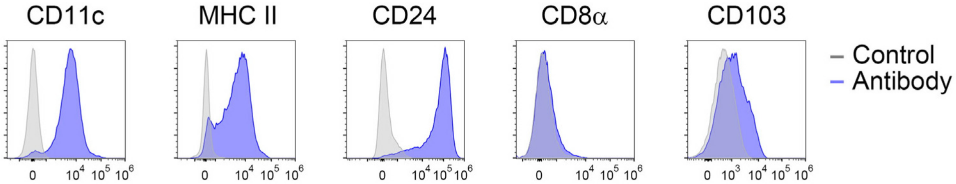

Supplementary Figure 4. Characterization of BMDCs. BMDCs were generated by FLT3-L. The expressions of CD11c, MHC II, CD24, CD8α, and CD103 were evaluated by flow cytometry. Shown are representative of two independent experiments.

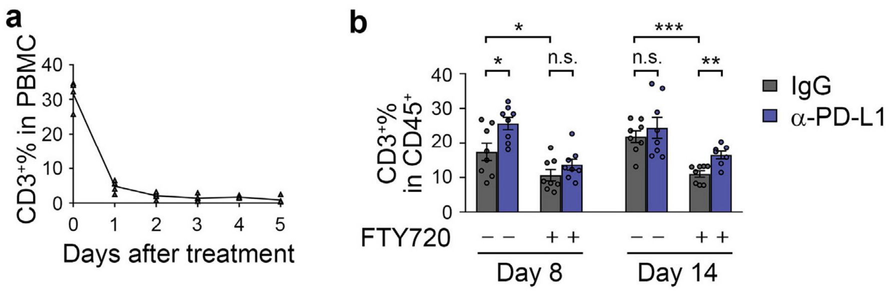

Supplementary Figure 5. Efficacy of FTY720 treatment. Mice were treated with FTY720 as in **Figure 5a**-**5c**. Percentages of T cells in PBMC (n = 5 mice, except day 0 n = 4) (**a**) and tumor tissues (n = 8 mice, except day 14 FTY720+anti-PD-L1 n = 7) (**b**) were shown. $*_{(8:\ IgG\ vs\ anti\text{-}PD\text{-}L1)}$p=0.0192; **p=0.0022; $*_{(8:\ IgG\ vs\ FTY720+IgG)}$p=0.0416; ***p<0.0001. Data are shown as mean ± SEM and are representative of three (**a**) or pool of two (**b**) independent experiments. n.s., not significant; *, p<0.05; **, p<0.01 determined by unpaired two-tailed Student's t-test in (**b**). Source data are provided as a Source Data file.

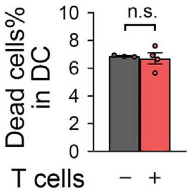

Supplementary Figure 6. T cell-mediated cytotoxicity is contact-dependent. Isolated DCs were loaded with OT-1 peptide. Activated OT-1 T cells were seeded on the upper side of a transwell membrane, while DCs were seeded on the lower side. Cell death of DCs was measured 4 hours later (n = 3 -T cells, 4 +T cells). Data are shown as mean ± SEM and are representative of two independent experiments. n.s., not significant determined by unpaired two-tailed Student's t-test. Source data are provided as a Source Data file.

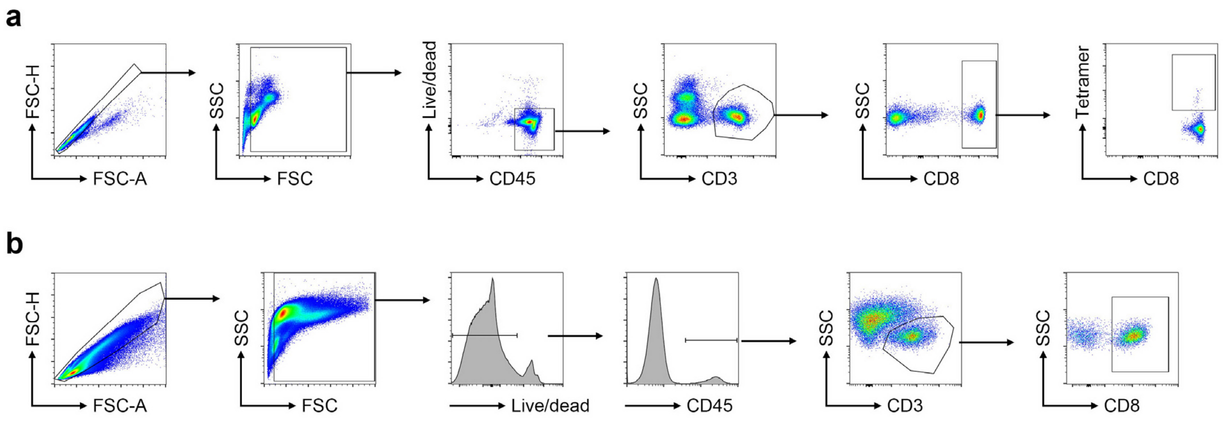

Supplementary Figure 7. Gating strategies used in the study. (**a**) PBMCs were gated for single live CD45$^+$ cells. Tetramer$^+$ cells were identified from CD8$^+$ T (CD3$^+$CD8$^+$) cells (Fig. 1f). (**b**) Gating strategy for CD8$^+$ T (CD3$^+$CD8$^+$) cells from tumor tissues (Fig. 5c).

Supplementary Table 1. List of antibodies and reagents used.

Reagent	Source	Identifier	Dilution
**Antibodies**			
anti-mouse CD103 FITC (clone 2E7)	eBioscience	11-1031-82	1:200
anti-mouse CD11b PE/Cy7 (clone M1/70)	eBioscience	25-0112-82	1:200
anti-mouse CD11c Alexa Fluor 700 (clone N418)	eBioscience	56-0114-82	1:200
anti-mouse CD24 PE (clone M1/69)	eBioscience	12-0242-82	1:200
anti-mouse CD3e PE/Cy7 (clone 145-2C11)	eBioscience	25-0031-82	1:200
anti-mouse CD45 FITC (clone 30-F11)	eBioscience	11-0451-85	1:200
anti-mouse CD45 PE (clone 30-F11)	eBioscience	12-0451-83	1:200
anti-mouse CD62L PE (clone MEL-14)	eBioscience	12-0621-82	1:200
anti-mouse CD8a Alexa Fluor 700 (clone 53-6.7)	eBioscience	56-0081-82	1:200
anti-mouse IFN-g PerCP/Cy5.5 (clone XMG1.2)	eBioscience	45-7311-82	1:50
anti-mouse MHC II APC (clone M5/114.15.2)	eBioscience	17-5321-82	1:200
anti-mouse MHC II PE/Cy5 (clone M5/114.15.2)	eBioscience	15-5321-82	1:200
anti-mouse PD-L1 APC (clone MIH5)	eBioscience	17-5982-82	1:200
anti-mouse PD-L1 PE (clone MIH5)	eBioscience	12-5982-83	1:200
anti-mouse CD11b biotin (clone M1/70)	BioLegend	101204	1:200
anti-mouse CD11c biotin (clone N418)	BioLegend	117304	1:200
anti-mouse CD19 Pacific Blue (clone 6D5)	BioLegend	115526	1:200
anti-mouse CD24 Pacific Blue (clone M1/69)	BioLegend	101820	1:200
anti-mouse CD4 APC/Cy7 (clone RM4-5)	BioLegend	100526	1:200
anti-mouse CD44 APC (clone IM7)	BioLegend	103012	1:200
anti-mouse CD45 Pacific Blue (clone 30-F11)	BioLegend	103126	1:200
anti-mouse CD8a APC/Cy7 (clone 53-6.7)	BioLegend	100714	1:200
anti-mouse CD8a PE (clone 53-6.7)	BioLegend	100708	1:200
anti-mouse F4/80 PE (clone BM8)	BioLegend	123110	1:200
anti-mouse Gr-1 FITC (clone RB6-8C5)	BioLegend	108406	1:200
anti-mouse IFN-g APC (clone XMG1.2)	BioLegend	505810	1:50
7-AAD	eBioscience	00-6993-50	1:20
Fixable Viability Dye eFluor 506	eBioscience	65-0866-14	1:1000
PI	Leagene	DA0028	1:20
Tetramer-SIINFEKL-APC	MBL	TS-5001-2C	1:20
Anti-CD16/32 (clone 2.4G2)	in house	N/A	
Anti-PD-L1 (Atezolizumab)	in house	N/A	
anti-mouse CD28 (clone 37.51)	BioXCell	BE0015	
anti-mouse CD3e (clone 145-2C11)	BioXCell	BE0001	
anti-mouse CD8a (clone YTS 169.4)	BioXCell	BE0117	
anti-mouse IFNAR-1 (clone MAR1-5A3)	BioXCell	BE0241	

anti-mouse IFN-g (clone XMG1.2)	BioXCell	BE0055	
anti-mouse PD-L1 (clone 10F.9G2)	BioXCell	BE0101	
**Chemicals, Peptides, and Recombinant Proteins**			
Recombinant murine FLT3-L	Sino Biological	51113-M02H	
Recombinant murine IFN-a	in house	N/A	
Recombinant murine IFN-g	Sangon Biotech	C600059	
Collagenase, Type IV	Invitrogen	17104019	
DNase I	Sigma	DN25	
FTY720	Cayman Chemical	10006292	
MTT	Sangon Biotech	A600799	
Puromycin	Solarbio	P8230	
SIY peptide (SIYRYYGL)	GL Biochem	N/A	
OT-1 peptide (SIINFEKL)	GL Biochem	N/A	
**Critical Commercial Assays**			
Mouse CD11c Positive Selection Kit II	STEMCELL	18780	
Mouse CD8+ T Cell Isolation Kit	STEMCELL	19853	
Mouse IFN-g ELISPOT Sets	BD Biosciences	551083	
