# Supplementary material for: PD-L1 on dendritic cells attenuates T cell activation and regulates response to immune checkpoint blockade
Source: Nat Commun. 2020 Sep 24;11:4835. doi: 10.1038/s41467-020-18570-x (PMC7518441; doi:10.1038/s41467-020-18570-x)
Supplement: Supplementary file 3 — Reporting Summary [file 41467_2020_18570_MOESM3_ESM.pdf]

nature research

Corresponding author(s): Haidong Tang

Last updated by author(s): Aug 21, 2020

Reporting Summary

Nature Research wishes to improve the reproducibility of the work that we publish. This form provides structure for consistency and transparency in reporting. For further information on Nature Research policies, see our Editorial Policies and the Editorial Policy Checklist.

Statistics

For all statistical analyses, confirm that the following items are present in the figure legend, table legend, main text, or Methods section.

n/a	Confirmed	
☐	☒	The exact sample size (*n*) for each experimental group/condition, given as a discrete number and unit of measurement
☐	☒	A statement on whether measurements were taken from distinct samples or whether the same sample was measured repeatedly
☐	☒	The statistical test(s) used AND whether they are one- or two-sided<br>*Only common tests should be described solely by name; describe more complex techniques in the Methods section.*
☒	☐	A description of all covariates tested
☒	☐	A description of any assumptions or corrections, such as tests of normality and adjustment for multiple comparisons
☐	☒	A full description of the statistical parameters including central tendency (e.g. means) or other basic estimates (e.g. regression coefficient) AND variation (e.g. standard deviation) or associated estimates of uncertainty (e.g. confidence intervals)
☐	☒	For null hypothesis testing, the test statistic (e.g. *F*, *t*, *r*) with confidence intervals, effect sizes, degrees of freedom and *P* value noted<br>*Give P values as exact values whenever suitable.*
☒	☐	For Bayesian analysis, information on the choice of priors and Markov chain Monte Carlo settings
☒	☐	For hierarchical and complex designs, identification of the appropriate level for tests and full reporting of outcomes
☒	☐	Estimates of effect sizes (e.g. Cohen's *d*, Pearson's *r*), indicating how they were calculated

Our web collection on statistics for biologists contains articles on many of the points above.

Software and code

Policy information about availability of computer code

Data collection	CytExpert 2.3; ImmunoSpot 5.1; Image Lab 5.2.1.
Data analysis	FlowJo v10.0.7; GraphPad Prism 8; Microsoft Excel 2016.

For manuscripts utilizing custom algorithms or software that are central to the research but not yet described in published literature, software must be made available to editors and reviewers. We strongly encourage code deposition in a community repository (e.g. GitHub). See the Nature Research guidelines for submitting code & software for further information.

Data

Policy information about availability of data

All manuscripts must include a data availability statement. This statement should provide the following information, where applicable:

- Accession codes, unique identifiers, or web links for publicly available datasets
- A list of figures that have associated raw data
- A description of any restrictions on data availability

The data supporting the findings of this study are available within the article and its Supplementary Information files and from the corresponding authors on reasonable request. The source data underlying Figs 1a, 1d-1g, 2a-2b, 3b-3g, 4a, 4c-4g, 5a-5f, and Supplementary Figs 1a-1f, 2a-2c, 3a-3b, 3d-3e, 5a-5b, and 6 are provided as a Source Data file.

Field-specific reporting

Please select the one below that is the best fit for your research. If you are not sure, read the appropriate sections before making your selection.

☒ Life sciences ☐ Behavioural & social sciences ☐ Ecological, evolutionary & environmental sciences

For a reference copy of the document with all sections, see nature.com/documents/nr-reporting-summary-flat.pdf

Life sciences study design

All studies must disclose on these points even when the disclosure is negative.

Sample size	A reasonable sample size was estimated from similar experiments in others' and our former publications (PMID: 29337303, 30389912, 26977880) to ensure adequate reproducibility of results. The exact n values used to calculate the statistics are provided.
Data exclusions	No data were excluded from the analyses.
Replication	All experiments were replicated for at least two times with similar results.
Randomization	Mice were randomly allocated into each group. For cell culture experiments, all treatments and controls were done together. Wells were randomly assigned for treatments.
Blinding	Collection of animal samples were not blinded when comparing naive vs tumor-bearing mice due to the obvious differences between groups (no tumor vs tumor-bearing). For other experiments, investigators were blinded to the genotype or treatment during data collection. For cell culture experiments, investigators were not blinded to group allocation, but had no vested interest in the conclusion. The samples were processed simultaneously or in parallel in all experiments. Data analysis was performed by a second person who was unaware of the groupings.

Reporting for specific materials, systems and methods

We require information from authors about some types of materials, experimental systems and methods used in many studies. Here, indicate whether each material, system or method listed is relevant to your study. If you are not sure if a list item applies to your research, read the appropriate section before selecting a response.

Materials & experimental systems

n/a	Involved in the study	
☐	☒	Antibodies
☐	☒	Eukaryotic cell lines
☒	☐	Palaeontology and archaeology
☐	☒	Animals and other organisms
☒	☐	Human research participants
☒	☐	Clinical data
☒	☐	Dual use research of concern

Methods

n/a	Involved in the study	
☒	☐	ChIP-seq
☐	☒	Flow cytometry
☒	☐	MRI-based neuroimaging

Antibodies

Antibodies used

Antibodies Source Identifier Application
anti-mouse CD103 FITC (clone 2E7) eBioscience 11-1031-82
anti-mouse CD11b PE/Cy7 (clone M1/70) eBioscience 25-0112-82
anti-mouse CD11c Alexa Fluor 700 (clone N418) eBioscience 56-0114-82
anti-mouse CD24 PE (clone M1/69) eBioscience 12-0242-82
anti-mouse CD3e PE/Cy7 (clone 145-2C11) eBioscience 25-0031-82
anti-mouse CD45 FITC (clone 30-F11) eBioscience 11-0451-85
anti-mouse CD45 PE (clone 30-F11) eBioscience 12-0451-83
anti-mouse CD62L PE (clone MEL-14) eBioscience 12-0621-82
anti-mouse CD8a Alexa Fluor 700 (clone 53-6.7) eBioscience 56-0081-82
anti-mouse IFN-g PerCP/Cy5.5 (clone XMG1.2) eBioscience 45-7311-82
anti-mouse MHC II APC (clone M5/114.15.2) eBioscience 17-5321-82
anti-mouse MHC II PE/Cy5 (clone M5/114.15.2) eBioscience 15-5321-82
anti-mouse PD-L1 APC (clone MIH5) eBioscience 17-5982-82
anti-mouse PD-L1 PE (clone MIH5) eBioscience 12-5982-83
anti-mouse CD11b biotin (clone M1/70) BioLegend 101204
anti-mouse CD11c biotin (clone N418) BioLegend 117304
anti-mouse CD19 Pacific Blue (clone 6D5) BioLegend 115526
anti-mouse CD24 Pacific Blue (clone M1/69) BioLegend 101820

anti-mouse CD4 APC/Cy7 (clone RM4-5) BioLegend 100526
anti-mouse CD44 APC (clone IM7) BioLegend 103012
anti-mouse CD45 Pacific Blue (clone 30-F11) BioLegend 103126
anti-mouse CD8a APC/Cy7 (clone 53-6.7) BioLegend 100714
anti-mouse CD8a PE (clone 53-6.7) BioLegend 100708
anti-mouse F4/80 PE (clone BM8) BioLegend 123110
anti-mouse Gr-1 FITC (clone RB6-8C5) BioLegend 108406
anti-mouse IFN-g APC (clone XMG1.2) BioLegend 505810
Tetramer-SIINFEKL-APC MBL TS-5001-2C
Anti-CD16/32 (clone 2.4G2), in house N/A
Anti-PD-L1 (Atezolizumab) in house N/A
anti-mouse CD28 (clone 37.51) BioXCell BE0015
anti-mouse CD3e (clone 145-2C11) BioXCell BE0001
anti-mouse CD8a (clone YTS 169.4) BioXCell BE0117
anti-mouse IFNAR-1 (clone MAR1-5A3) BioXCell BE0241
anti-mouse IFN-g (clone XMG1.2) BioXCell BE0055
anti-mouse PD-L1 (clone 10F.9G2) BioXCell BE0101

Validation

In house produced anti-PD-L1 antibody has been validated by flow cytometry staining wild-type vs PD-L1-/- cell line. Commercial antibodies used have been validated by the vendors and validation data are available on the manufacturer's website. All commercial antibodies have been further validated with flow cytometric analysis of target expression on mouse splenocytes in our lab.

Eukaryotic cell lines

Policy information about cell lines

Cell line source(s)

MC38, E.G7, B16F10, and 293T cells were from American Type Culture Collection (ATCC).

Authentication

No authentication was carried out. Cell morphology and behavior such as tumor growth and response to immune checkpoint blockade therapy was consistent with expectations.

Mycoplasma contamination

Cell lines were tested negative for mycoplasma contamination.

Commonly misidentified lines
(See ICLAC register)

No misidentified lines were used.

Animals and other organisms

Policy information about studies involving animals; ARRIVE guidelines recommended for reporting animal research

Laboratory animals

Wild-type C57BL/6 mice were purchased from Vital River (Beijing, China). Batf3-/- and CD11c-cre mice were purchased from the Jackson Laboratory. Pdl1fl/fl mice were generated in the animal core. All mice were maintained under specific pathogen-free conditions at 22-26 oC with a 12:12 hour dark/light cycle and 40-70% humidity.
Wild-type female mice were used at an age of 6-8 weeks. For genetic modified mice, age and sex matched mice were used for each experiment. Both female and male mice were used at an age of 6-12 weeks.

Wild animals

The study did not involve wild animals.

Field-collected samples

The study did not involve samples collected from the field.

Ethics oversight

Animal experiment protocols were consistent with guidelines of the Laboratory Animal Research Center of Tsinghua University. All animal studies were approved by the Animal Care and Use Committee of Tsinghua University.

Note that full information on the approval of the study protocol must also be provided in the manuscript.

Flow Cytometry

Plots

Confirm that:

☒ The axis labels state the marker and fluorochrome used (e.g. CD4-FITC).

☒ The axis scales are clearly visible. Include numbers along axes only for bottom left plot of group (a 'group' is an analysis of identical markers).

☒ All plots are contour plots with outliers or pseudocolor plots.

☒ A numerical value for number of cells or percentage (with statistics) is provided.

Methodology

Sample preparation

Tissues (tumor, lymph node, and spleen) were cut into small pieces before digested in RPMI-1640 medium with 1 mg/ml type IV collagenase and 100 ug/ml DNase I. After digestion, tissues were passed through a 70 um cell strainer to make single cell

	suspensions. Cells were blocked with anti-CD16/32 (clone 2.4G2) for 20 min at room temperature. Then cells were incubated with antibodies for 30 min. After washed, samples were analyzed on a flow cytometer.
Instrument	CytoFLEX S (Beckman Coulter)
Software	CytExpert 2.3; FlowJo v10.0.7.
Cell population abundance	For flow sorting, post-sort cells were analyzed by flow cytometry and the purity was at least 95%. For MACS sorting, post-sort cells were at least 95% pure for CD8 T cells and 80% pure for DC.
Gating strategy	FSC/SSC were gated based on cell sizes comparing to a standard beads. Posive/negative boundaries were determined by comparing with an FMO (fluoresent minus one) control. A sample gating strategy is provided in supplementary figure.

☒ Tick this box to confirm that a figure exemplifying the gating strategy is provided in the Supplementary Information.
